# Dermoscopy of Basal Cell Carcinoma Part 1: Dermoscopic Findings and Diagnostic Accuracy—A Systematic Literature Review

**DOI:** 10.3390/cancers17030493

**Published:** 2025-02-01

**Authors:** Irena Wojtowicz, Magdalena Żychowska

**Affiliations:** Department of Dermatology, Faculty of Medicine, Medical College of Rzeszow University, 35-310 Rzeszow, Poland

**Keywords:** dermoscopy, dermatoscopy, basal cell carcinoma, polarized, non-polarized

## Abstract

Introduction: Basal cell carcinoma (BCC) is the most common malignant skin tumor. While rarely fatal, it can cause local tissue damage. Part I of the review summarizes the dermoscopic features of BCC and the diagnostic accuracy of dermoscopy in the diagnosis of BCC. Methods: A search of the PubMed database was performed for studies reporting on the diagnostic accuracy of dermoscopy or dermoscopic findings in BCC, either pigmented or non-pigmented, located anywhere on the body, of any histopathologic subtype, size and at any age of onset. Results: BCC was found to present with a wide range of dermoscopic features, including white structures (shiny white lines, shiny white areas, rosettes), yellow structures (milia-like cysts, yellow lobular-like structures), multiple aggregated yellow-white globules (MAY globules), blue structures (blue ovoid nests), vascular structures (arborizing vessels, short fine telangiectasias), multiple small erosions/ulcerations, features of regression (pepper-like structures, white scar-like areas) and pigmented structures (spoke-wheel areas, maple leaf-like areas (MLLAs), blue/gray dots). Dermoscopy showed a sensitivity of 67.6–98.6% and a positive predictive value (PPV) of 85.9–97% in identifying BCC. The physician’s experience and training improve the accuracy, however, BCCs on the trunk and extremities, particularly of superficial subtypes, may still constitute a challenge. Conclusions: Dermoscopy, especially when performed by a trained physician, increases the accuracy of early BCC detection.

## Simple Summary

Basal cell carcinoma (BCC) is the most common malignant skin tumor, which can cause significant tissue damage. BCC may present with a wide range of dermoscopic findings, including white, yellow, blue and pigmented structures, vascular structures (predominantly arborizing vessels or short fine teleangiectasias), multiple small erosions/ulcerations and/or features of regression. Dermoscopy improves the diagnostic accuracy of early BCC detection when compared with clinical examination alone. Diagnostic accuracy also increases with training and experience, which constitutes the premise for the need to expand dermoscopic knowledge.

## 1. Introduction

Basal cell carcinoma (BCC) is the most common malignant skin tumor, accounting for 75–90% of skin cancers and occurring 18–20 times more often than melanoma. It is also the most prevalent malignant disease overall [1,2,3]. About one in five people will develop BCC at some point in life, with a higher incidence in men [4,5].

BCC grows slowly, usually less than 1 mm^2^ per month, and while rarely fatal or metastatic, it can cause significant local tissue damage [6,7,8]. Dermoscopy is a noninvasive method that visualizes skin features from the surface to the papillary dermis and facilitates appropriate lesion management [1,9].

Part I of the systematic review summarizes the dermoscopic features of BCC and the diagnostic accuracy of dermoscopy in the diagnosis of BCC.

## 2. Methods

**Figure 1:**
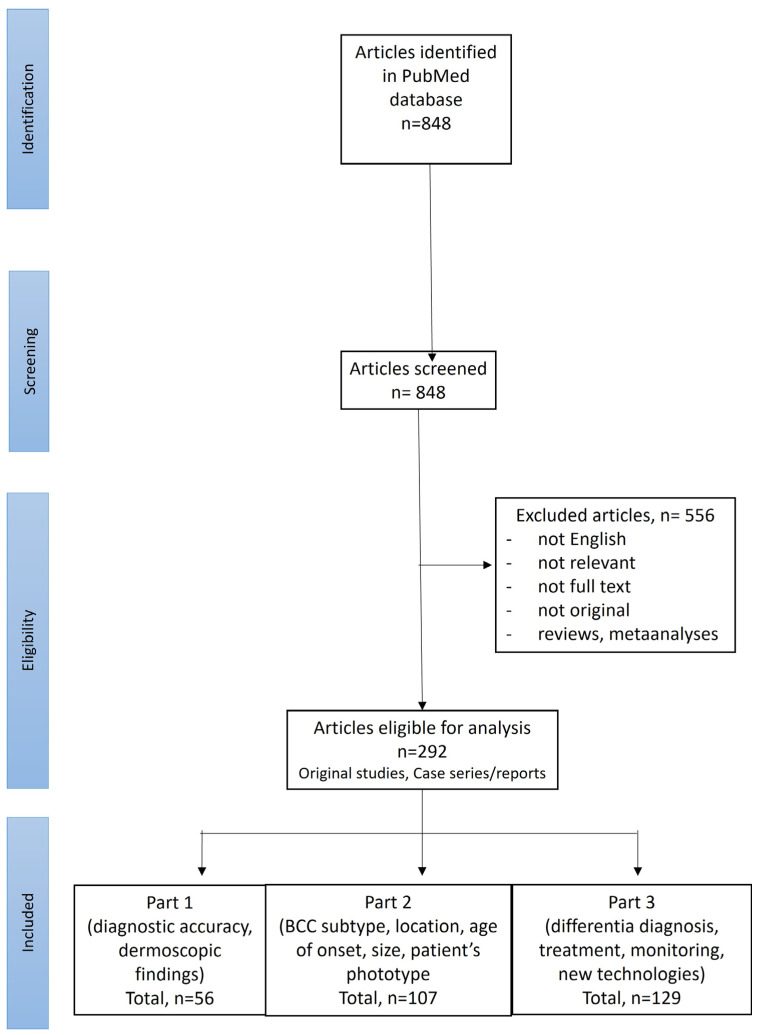
PRISMA flow chart showing the screening process (available also: Wojtowicz I et al. [10]).

A search of the PubMed database was performed using the terms “(BCC OR basal cell carcinoma OR basalioma) AND (dermoscopy OR dermatoscopy)”. All records from the establishment of the PubMed database through September 2024 were analyzed. Only English-language publications were included. Additionally, the reference lists of all the reports were checked for relevant articles. Two reviewers (I.W. and M.Ż.) initially checked the titles and abstracts of all of the records. If a title or abstract suggested the study might be relevant, the full article was reviewed. The criteria for including studies were: (1) Patients with histopathologically confirmed BCC who underwent a dermoscopic examination; (2) The studies reported on the diagnostic accuracy of dermoscopy, dermoscopic findings of BCC, both pigmented or non-pigmented, located anywhere on the body, of any histopathologic subtype, size and at any age of onset. Figure 1 shows the process of selecting articles based on the PRISMA (Preferred Reporting Items for Systematic Reviews and Meta-Analyses) guidelines.

## 3. Results

The literature search initially identified 848 studies. After screening abstracts, 292 articles were selected for further review. Of these, 56 articles reported on the diagnostic accuracy of dermoscopy or characterized dermoscopic features of BCC and were included in Part 1 of the review.

### 3.1. Diagnostic Accuracy of Dermoscopy

Several studies assessed the diagnostic accuracy of dermoscopy in BCC, with most being retrospective and conducted in Western countries. A large Swedish study analyzing 1180 histologically confirmed BCCs found that diagnosis with dermoscopic evaluation had a sensitivity of 95.4% and a positive predictive value (PPV) of 85.9% [11]. Similar results were reported by Altamura et al., with an even higher PPV of up to 96% [12]. Nelson et al. reported a sensitivity of 67.6% and a PPV of 97.0%, although the lower sensitivity was explained by the study’s design, which assessed the decision to refer patients directly for definitive treatment without an incisional biopsy [13]. Dermoscopy was shown to have a specificity of 100%, sensitivity of 80%, PPV of 100% and a negative predictive value (NPV) of 94.12% for identifying malignant lesions [14].

Dermoscopic accuracy for BCC diagnosis was reported to be higher in case of pigmented lesions [1,6]. An analysis of 934 BCCs in a Japanese population, of which 96.4% were pigmented, showed that the diagnostic sensitivity and specificity were 92.2% and 96.0%, respectively [6]. Rosendahl et al. demonstrated a sensitivity of 98.6% for pigmented BCCs. The authors also found that adding dermoscopy to clinical examination improved diagnostic accuracy for pigmented lesions, but this improvement was statistically significant only for nonmelanocytic lesions [15].

In a multicenter prospective study on 740 BCCs, the sensitivity and specificity of dermoscopy were 93.2% and 51.7%, with a PPV of 84.4% and an NPV of 73.3%. The lower specificity was explained by the high number (87%) of hypopigmented/amelanotic lesions in the study group [1]. Low specificity (51.8% and 69.5% for two observers) was also shown by Guitera et al., who recruited to their study only amelanotic and light-colored lesions [16]. The study of ‘pink’ cutaneous lesions resulted in an overall BCC sensitivity of 85.1% and specificity of 92.4%, with a PPV of 89.8% [17].

Yuki et al. found that sensitivity was significantly lower for BCCs located on the trunk and extremities, attributing this to alower frequency of pigmentation and a higher proportion of superficial BCCs in those areas [6].

Another factor influencing diagnostic accuracy by dermoscopy was the doctor’s experience. Nelson et al. showed that gaining expertise resulted in higher sensitivity at the cost of specificity in direct referral to surgery [12]. Yuki et al. also observed that sensitivity increased with experience, reaching a plateau after seven months [6]. The most common misdiagnosis for BCC was seborrheic keratosis, followed by melanocytic nevus [6].

Some studies suggested that dermoscopy can predict histopathologic subtypes with up to 93.3% accuracy, with the highest value for superficial and nodular variants [18,19,20,21]. However, others did not confirm these results, especially in cases of aggressive variants of BCC [13,22,23]. Popadić and Brasanac concluded that dermoscopy does not accurately reflect histopathologic findings in aggressive BCCs [19].

### 3.2. Dermoscopy Findings

A wide range of dermoscopic findings in BCC have been reported in the literature and, taking into account common features, they were divided into the following groups: white structures (shiny white lines, shiny white areas, rosettes), yellow structures (milia-like cysts, yellow lobular-like structures), MAY globules, blue structures (blue ovoid nests), vascular structures (predominantly arborizing vessels or short fine teleangiectasias), multiple small erosions/ulcerations, features of regression (“blue areas”, “blue hue”, “pepper-like structures”, “white scar-like areas”, “white areas”, “milky way areas”) and pigmented structures (spoke-wheel areas, maple leaf-like areas–MLLAs, large blue-gray ovoid nests and less frequently multiple blue/gray globules, multiple in-focus blue/gray dots, blue-whitish veils, brown dots or globules, concentric structures, pigment networks). Several novel findings, such as negative maple leaf-like areas (NMLLAs), brown homogenous blotches, large blue-gray structureless areas, interrupted radial streaking, rainbow patterns and semitranslucent areas, are also discussed below.

#### 3.2.1. White Structures

White structures observed in the dermoscopy of BCC include shiny white lines, shiny white areas (blotches) and rosettes. A key feature is that these structures are visible only under polarized light (contact or non-contact) and are not seen with non-polarized dermoscopy [24,25,26,27,28].

Shiny white lines, described as bright whitish lines, were initially referred to as ‘chrysalis structures’, though this term was considered a misnomer. The preferred term now is ‘crystalline structures’ or ‘crystalline lines’. They include both short lines and longer strands [28]. Apart from BCCs, they can be observed in various malignant and benign lesions, including melanoma, squamous cell carcinoma (SCC), lichen planus-like keratosis (LPLK), Spitz nevi, scars, dermatofibromas, porokeratosis and even on extensively sun-damaged skin of the bald scalp [25,26,27,28,29].

In a prospective observational study analyzing 11,225 lesions, Balagula et al. found that only 1.8% of lesions presented shiny white lines. These lines were primarily seen in dermatofibromas (75.2%) and scars (90.5%), but also in BCC (47.6%) and invasive melanomas (84.6%), and rarely in nevi. The study demonstrated that these structures were 2.5 times more likely to be observed in malignant tumors compared to benign lesions [29]. In another study Shitara et al. concluded that the presence of shiny white lines is associated with a ten times higher risk of malignancy [25].

Navarrete-Dechent et al. found that shiny white strands and/or blotches (white clods or larger structureless areas) had a diagnostic specificity of 91% and should be considered a reliable criterion for detecting BCC, although the study’s limitation was that it included only non-pigmented neoplasms [26]. Liebman et al. confirmed that the presence of these structures is suggestive of a BCC [28]. To date, no statistically significant difference in the prevalence of these structures across different histopathological subtypes of BCC has been reported [24,25,26]. However, shiny white strands are considered to be more frequently present in BCCs with ulceration [25].

According to Liebman et al., in BCC, larger whitish strands were observed more often than short lines (41% vs. 12%), and they were typically arranged in parallel or disorganized rather than orthogonally oriented (35.8% vs. 44.4% vs. 19.8%, respectively). In contrast, in melanomas, short shiny white lines were more frequently present and were typically arranged orthogonally, without accompanying shiny white areas [28].

**Figure 2:**
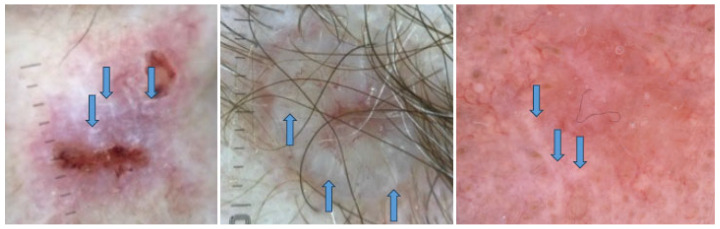
Dermoscopy images of basal cell carcinomas (BCCs) with shiny white lines (blue arrows).

**Figure 3:**
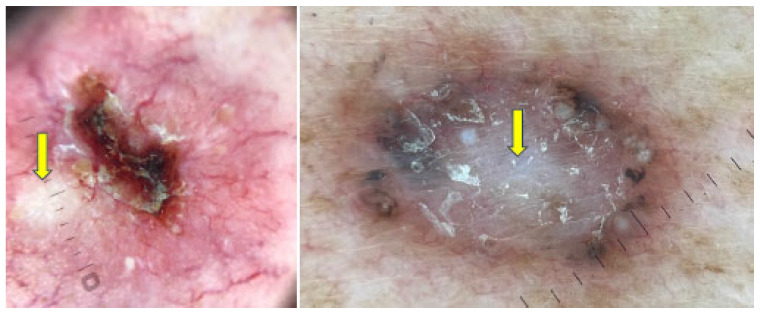
Dermoscopy images of BCCs with shiny white blotches (yellow arrows).

Examples of BCCs displaying shiny white lines are shown in Figure 2, while those with shiny white blotches are illustrated in Figure 3.

Rosettes, defined as four bright white points clustered together, are significantly more likely to be seen in actinic tumors than in other lesions [28]. Liebman et al. reported that 46.3% of actinic keratoses and 27% of SCCs showed rosettes [28]. Navarrete-Dechent et al. concluded that rosettes were not associated with a diagnosis of BCC [26].

A study from 1998 identified an additional whitish dermoscopic feature: a milky-red peripheral ring with intersecting blood vessels, referred to as “corona”. This feature was found in nodular and infiltrative BCC, but rarely in the superficial subtype [9]. However, this finding has not been reported in more recent studies.

#### 3.2.2. Yellow Structures

Yellowish structures observed in the dermoscopy of BCC include milia-like cysts (MLCs) and yellow lobular-like structures.

**Figure 4:**
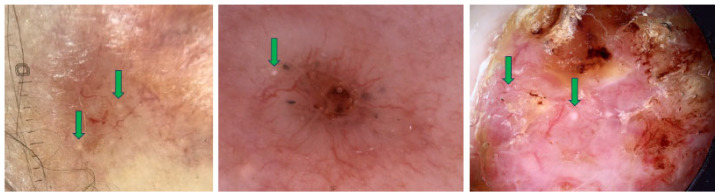
Dermoscopy images of BCCs with milia-like cysts (MLCs) (green arrows).

According to Bellucci et al., MLCs show starry and cloudy formations, which may also appear white in color. The starry formations are bright at the center with variably sharp borders, while the cloudy ones are larger, have fluffy borders and are approximately oval in shape. In an analysis of 400 BCCs, MLCs were present in 7.75% of cases [30]. MLCs are typically found in seborrheic keratoses (more often as cloudy MLCs) and congenital melanocytic nevi and were identified as useful features for differentiating benign lesions from melanoma [30]. They are more clearly visible under non-polarized dermoscopy [24]. Figure 4 shows examples of BCCs with MLCs.

**Figure 5:**
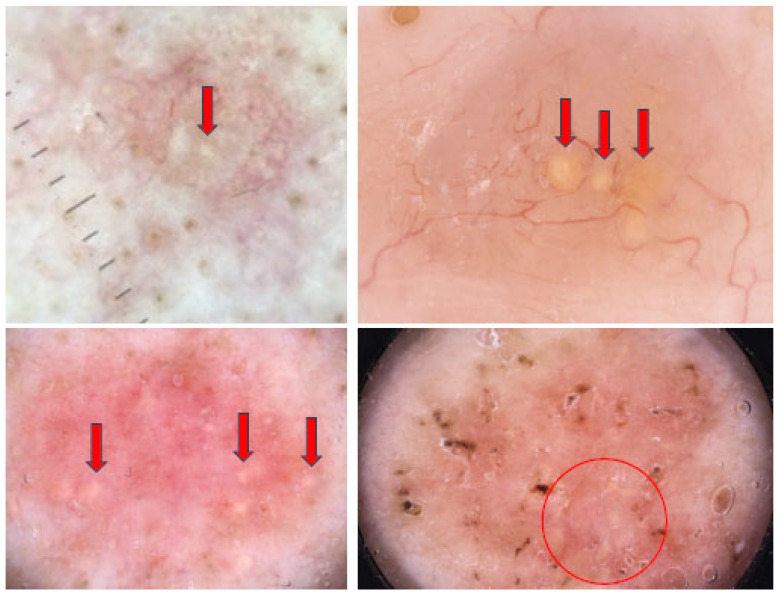
Dermoscopy images of BCCs with yellow lobular-like structures (red arrows and red circle).

Yellow lobular-like structures are round or oval in shape, vary in size and may be either isolated or clustered together. In the aforementioned study, they were noted in 4.2% of BCCs. Moreover, they have been thought to be characteristic for sebaceous hyperplasia, sebaceous adenoma and nevus sebaceous of Jadassohn [30]. Figure 5 illustrates BCCs with yellow lobular-like structures.

Notably, the study found that both types of yellow structures were more frequently seen in BCCs located on the face and in nodular subtypes. The authors concluded that their presence should not rule out a BCC diagnosis when other specific dermoscopic criteria are present [30].

In 2021, Roda and Oliveira reported a case of a “half-yellow BCC”. On histopathology, the yellow color corresponded to cholesterol clefts, which might have resulted from microtrauma or long-lasting disease [31].

#### 3.2.3. MAY Globules

**Figure 6:**
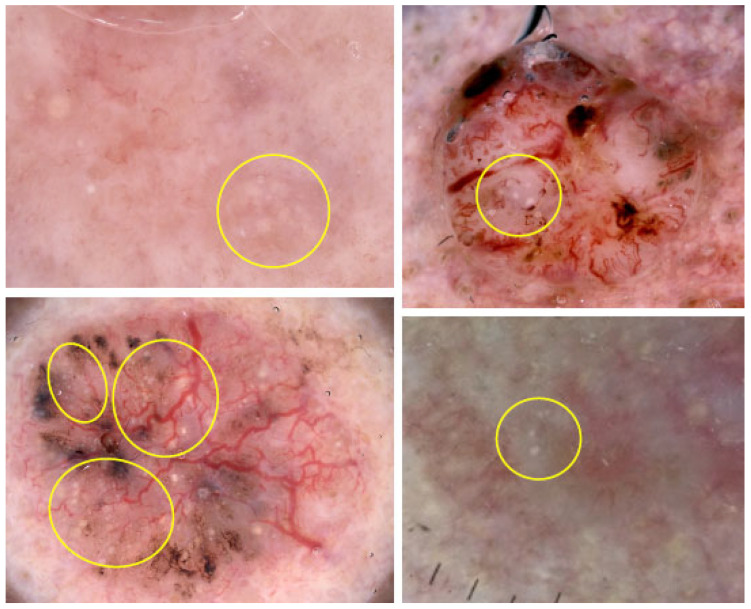
Dermoscopy images of BCCs with multiple aggregated yellow-white globules (MAY globules) (yellow circles).

MAY globules, defined as clustered white-yellow structures (multiple aggregated yellow-white globules)*,* are primarily associated with BCC. Some examples are present in Figure 6. These globules are visible under both polarized and non-polarized light [32,33]. In a case-control study on 656 non-pigmented lesions, MAY globules were found in 21.0% of BCCs, but were rare in other diagnoses (only 0.8% of cases), including SCC and desmoplastic trichoepithelioma. Among BCCs located on the head and neck, 38.7% exhibited MAY globules, compared to just 4.2% of lesions other than BCC. Their presence can be helpful in distinguishing BCC from intradermal nevi, effectively excluding the latter [34].

In the aforementioned study, MAY globules were significantly associated with high-risk histologic subtypes of BCC, such as infiltrative and morpheaform types, where they were 6.5 times more likely to occur. Notably, they were not observed in any case of superficial BCC [32].

In several cases, MAY globules were linked with calcifications observed in histopathology, supporting earlier findings that calcifications are more common in high-risk subtypes [32]. Pagnoni et al. reported a case of calcifying micronodular BCC with MAY globules and concluded that calcifications, as well as micronodular histopathologic changes, were associated with aggressive subtypes of BCC [33].

#### 3.2.4. Blue Structures

Under dermoscopy, a blue color has been a known indicator of malignancy. In 2017, Papadić et al. reported that nearly two-thirds of lesions with blue structures were likely to be malignant. In their analysis, BCCs constituted 21% of 144 pigmented lesions displaying a blue color under dermoscopy. The authors found the distribution of color to be crucial. Structureless peripheral or patchy blue color were most frequently observed in melanoma, while blue clods were more indicative of BCC. Among 28 lesions with blue clods, 17 (60.7%) were confirmed to be BCCs. These blue clods, metaphorically referred to as blue ovoid nests, were also observed in nevi (14.3%), seborrheic keratoses (7.14%) and angiomas (3.57%) [35].

#### 3.2.5. Vascular Structures

Vascular structures are crucial in the diagnosis of BCC, particularly in non-pigmented lesions, where the lack of characteristic pigmented features makes diagnosis more challenging. According to Micantonio et al., nearly all BCCs (91.5%, 461 out of 504) presented with at least one vascular pattern [36]. Sakakibara et al. reported a slightly lower but still significant incidence of 87% in the BCCs diagnosed in Japanese population [34]. Vessels are more clearly visible in non-contact dermoscopy due to the absence of pressure applied to the surface, which occurs in contact dermoscopy [24].

**Figure 7:**
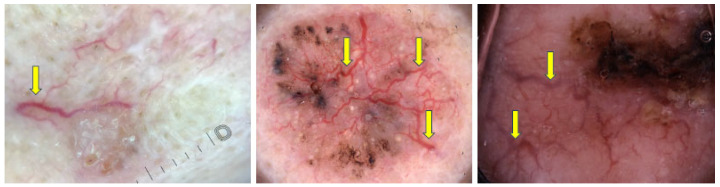
Dermoscopy images of BCCs with arborizing vessels (yellow arrows).

The two most common vascular patterns in BCC are arborizing vessels and short fine telangiectasias [24,37,38,39,40]. Arborizing vessels, defined as large stem vessels (≥0.2 mm) that branch irregularly in a tree-like pattern, were the most frequently observed in nodular and pigmented subtypes [36,40]. Gürsel et al. noted arborizing vessels as the most common BCC feature [38], with a sensitivity of 72% and specificity of 100% in the Japanese population [37]. Figure 7 presents dermoscopy images of these structures.

**Figure 8:**
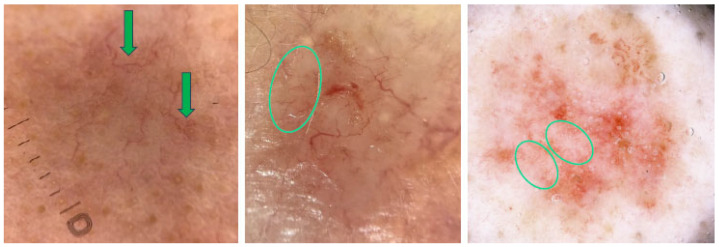
Dermoscopy images of BCCs with short fine telangiectasias (green arrows and circles).

Short fine telangiectasias are defined as fine, kinked vessels of small caliber and length with few branches [36]. They are observed in 10% of BCCs and are considered an early form of arborizing vessels [12]. These vessels are typical of superficial BCC, particularly observed in the non-pigmented subtype [34,36]. Figure 8 shows some BCCs with short fine telangiectasias.

Arpaia et al. analyzed the type and prevalence of vascular patterns in ulcerated and non-ulcerated portions of BCC. They found that dotted, linear-irregular, hairpin, comma and polymorphous patterns were highly represented in the ulcerated areas, while the arborizing vessels were prevalent in the non-ulcerated portion. Moreover, the correct diagnosis of BCC was statistically more likely when the ulcerated portion showed an arborizing pattern or when glomerular or hairpin patterns were absent. Additionally, the absence of dotted vessels in the non-ulcerated areas also increased the likelihood of an accurate diagnosis [34].

Additional vascular structures appear in less than 10% of BCCs and are almost always associated with arborizing vessels or short fine telangiectasias. These additional structures can include hairpin, glomerular, dotted, comma or polymorphous vessels, with decreasing incidence respectively. When a lesion contains two or more vascular patterns, it is referred to as polymorphous and needs further investigation to exclude amelanotic/hypomelanotic melanoma and SCC [36].

#### 3.2.6. Multiple Small Erosions/Ulcerations

**Figure 9:**
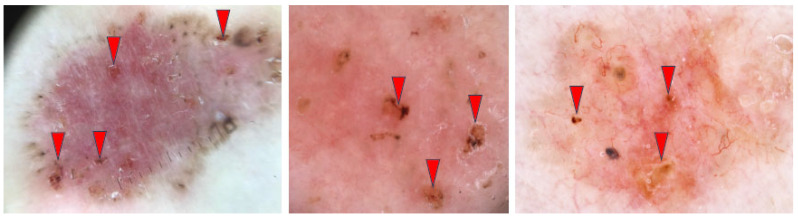
Dermoscopy images of BCCs with multiple small erosions/ulcerations (red arrowheads).

By definition, erosions are characterized by superficial tissue loss, whereas ulcerations involve deeper, full-thickness loss of the entire epidermis and superficial dermis [41,42]. The occurrence of multiple small erosions was reported to be the key dermoscopic feature that distinguished superficial BCC from other histopathological subtypes [43]. Figure 9 illustrates several BCCs with multiple small erosions/ulcerations.

#### 3.2.7. Features of Regression

**Figure 10:**
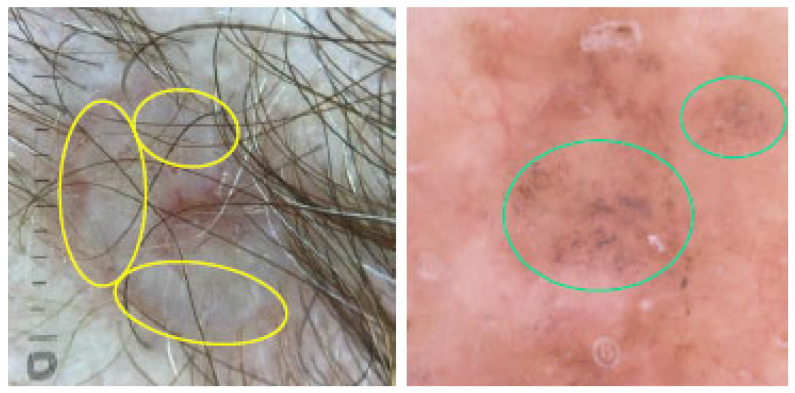
Dermoscopy images of BCCs with bluish features of regression called “blue areas” or “blue hue” (yellow circles) and “pepper-like structures” (green circles).

**Figure 11:**
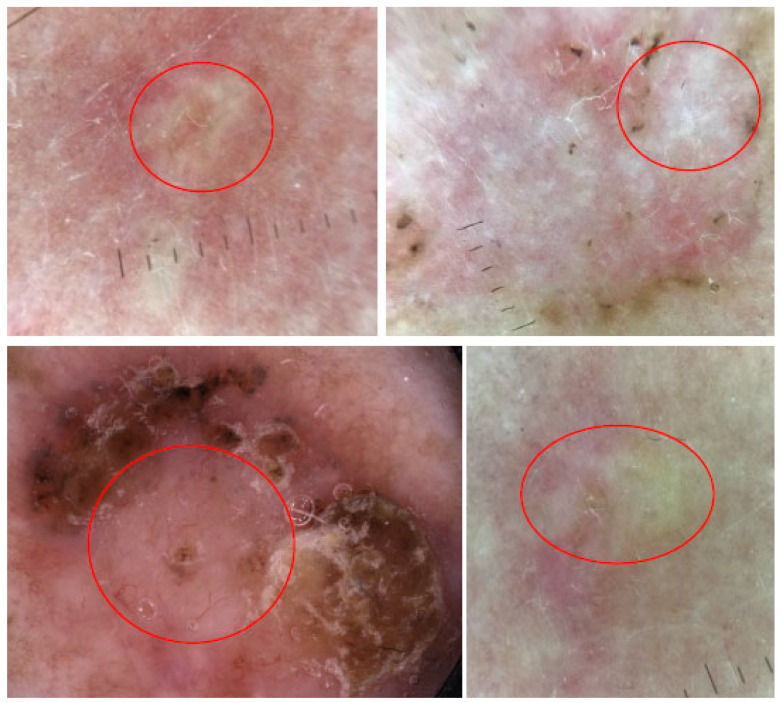
Dermoscopy images of BCCs with white/reddish features of regression called “white scar-like areas”, “white areas” or “milky way areas” (red circles).

In dermoscopy, features of regression may appear as bluish areas (due to the accumulation of melanin) or as white/reddish areas (indicating fibroplasia with the formation of blood vessels). The former, shown in Figure 10, are often referred to as “blue areas”, “blue hue” or “pepper-like structures”, while the latter, presented in Figure 11, are commonly called “white scar-like areas”, “white areas” or “milky way areas”. These features disrupt the overall dermoscopic presentation of the lesion, making the diagnosis more challenging. Features of regression are primarily considered to be a sign of melanoma. However, they may also be seen in many other lesions, including BCC, SCC, Bowen’s disease, pigmented actinic keratoses and seborrheic keratoses. Therefore, regression should not be considered an independent indicator of melanoma but rather assessed in combination with other dermoscopic features [44].

#### 3.2.8. Pigmented Structures

Diagnostic criteria for pigmented BCC were presented by Menzies et al. in 2000. They included an absence of pigment network and the presence of one or more of the following six features: spoke-wheel areas, MLLAs, large gray-blue ovoid nests, multiple gray-blue globules, ulceration and arborizing vessels [3,7,12,45]. The method was demonstrated to have a sensitivity of 97% for diagnosing pigmented BCCs, and a specificity of 93% for invasive melanoma and 92% for benign pigmented skin lesions [45].

Peris et al. assessed the interobserver agreement, with five observers with varying levels of dermoscopy experience, of each dermoscopic feature proposed by Menzies. Full agreement was reached for the absence of a pigment network. Spoke-wheel areas and arborizing vessels showed very good agreement, while ulceration and multiple blue-gray globules had good agreement. However, there was no agreement on the definitions of MLLAs and large blue-gray ovoid nests, as these structures were often confused with each other; ovoid nests may be misinterpreted as globules or MLLAs and the latter as ovoid nests or localized pigmentation [46].

The pigment network is the most characteristic feature of melanocytic lesions and generally should not be found in BCC. However, in a study analyzing 412 BCCs, pigment network or network-like structures were observed in 3.4% of BCCs. In 64.3% of these cases, the presence of the pigment network was due to the collision of BCC with another skin neoplasm, such as solar lentigo, nevus or actinic keratosis. Such findings may result from the lesion’s location on photodamaged skin and, most importantly, should always be carefully distinguished from atypical or malignant melanocytic lesions [7,47,48].

**Figure 12:**
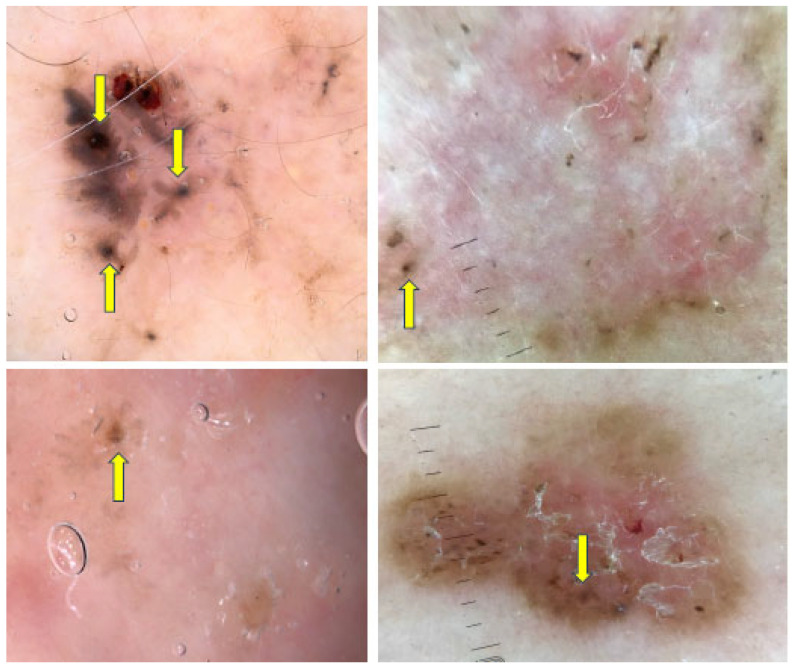
Dermoscopy images of BCCs with spoke-wheel areas (yellow arrows).

Spoke-wheel areas are well-circumscribed radial projections, typically brown but occasionally blue or gray, converging at a central axis that is often darker in color (e.g., dark brown, black or blue), as illustrated in Figure 12 [45]. They are considered to be highly specific indicator of pigmented BCC, with specificity reaching 100% in some studies [42,46,49,50]. Longo et al. demonstrated that the presence of spoke-wheel or concentric structures was the most significant factor in predicting a BCC diagnosis [1]. In a study analyzing BCC thickness, spoke-wheel areas were more limited in thicker tumors, while they covered wider areas in thinner tumors [4].

**Figure 13:**
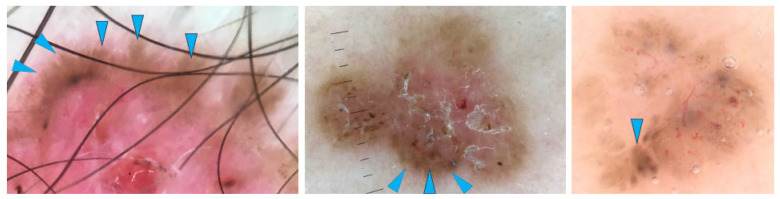
Dermoscopy images of BCCs with maple leaf-like areas—MLLAs (blue arrowheads).

MLLAs are brown to gray-blue bulbous extensions that create a leaf-like pattern. Some examples are shown in Figure 13. Unlike pseudopods, these areas are distinct pigmented nests (islands) that do not arise from a pigment network and generally do not arise from an adjacent confluent pigmented area [45]. MLLAs were more frequently observed in younger individuals compared to other BCC features and were more commonly found in lesions with smaller diameters, suggesting that they may be an early sign of pigmented BCC [51].

**Figure 14:**
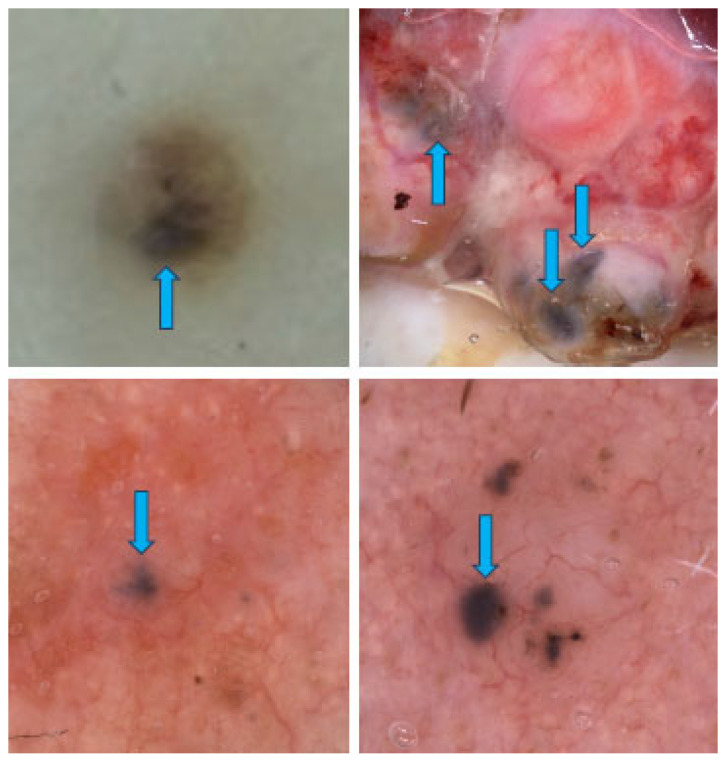
Dermoscopy images of BCCs with blue-gray ovoid nests (blue arrows).

Large blue-gray ovoid nests are well-defined pigmented areas (either ovoid or elongated) larger than globules that are confluent or nearly confluent and not directly connected to the main pigmented tumor body [45]. Figure 14 illustrates some of them. The presence of blue ovoid nests has been reported to be associated with increased BCC thickness, confirmed both by ultrasound and histopathology [4].

In a study conducted by Tabanlıoğlu et al., 57.5% of pigmented BCCs presented with a blue-whitish veil, suggesting that this feature might play a more significant role in identifying pigmented BCC than previously thought, as this finding has traditionally been associated with melanoma [51,52]. A blue-whitish veil is better seen in non-polarized dermoscopy [11].

Brown dots or globules may occasionally be observed in BCC; however, they are typically seen in melanocytic lesions [53].

**Figure 15:**
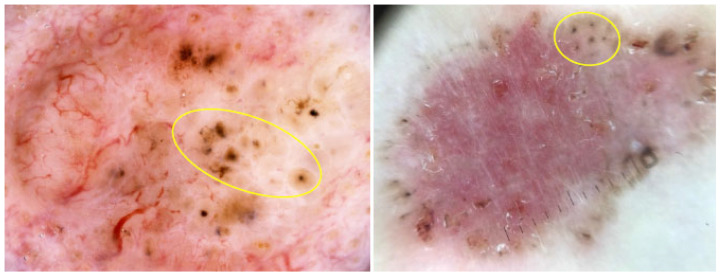
Dermoscopy images of BCCs with concentric structures (yellow circles).

**Figure 16:**
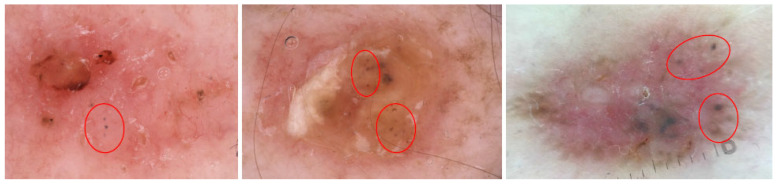
Dermoscopy images of BCCs with multiple blue/gray globules (red circles).

**Figure 17:**
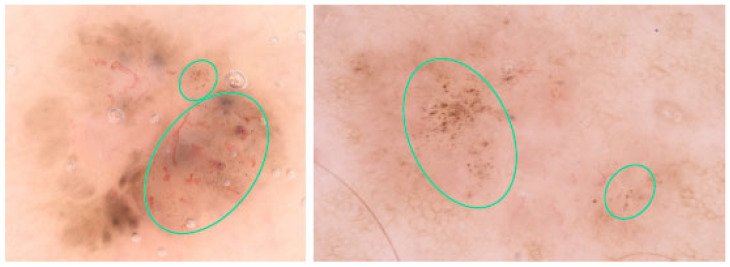
Dermoscopy images of BCCs with multiple in-focus blue/gray dots (green circles).

Concentric structures and multiple in-focus blue/gray dots were first reported by Altamura et al. and observed in 7.6% and 5.1% of the BCCs, respectively. The authors concluded that concentric structures represent the early stage of a spoke-wheel area, while multiple in-focus blue/gray dots indicate the early phase of multiple blue/gray globules [12]. Examples of BCCs with concentric structures, multiple blue-gray globules, and multiple in-focus blue-gray dots are presented in Figure 15, Figure 16 and Figure 17, respectively.

It is worth mentioning that the classic dermoscopic features of pigmented BCC (large blue/gray ovoid nests, multiple blue/gray globules and ulceration) can be disrupted by previous treatments with ablative lasers, which makes the diagnosis more challenging. Interestingly, arborizing vessels and certain nonclassical dermoscopic patterns (short fine superficial telangiectasia, multiple small erosions, concentric structures, multiple in-focus blue/gray dots) were found to be predominantly preserved after such procedures [54].

#### 3.2.9. Novel Findings

##### Negative Maple Leaf-like Areas

NMLLAs, described in 2024 by Palmisano et al., are a non-pigmented version of the MLLAs initially characterized by Menzies et al. in 2000 [2,45]. They are characterized by round, non-pigmented bulbous structures with a homogeneous whitish background and well-defined borders. This newly described feature is associated with superficial BCC and reflects non-pigmented tumor nests at the dermal-epidermal junction [2].

##### Brown Homogeneous Blotches (BHB)

**Figure 18:**
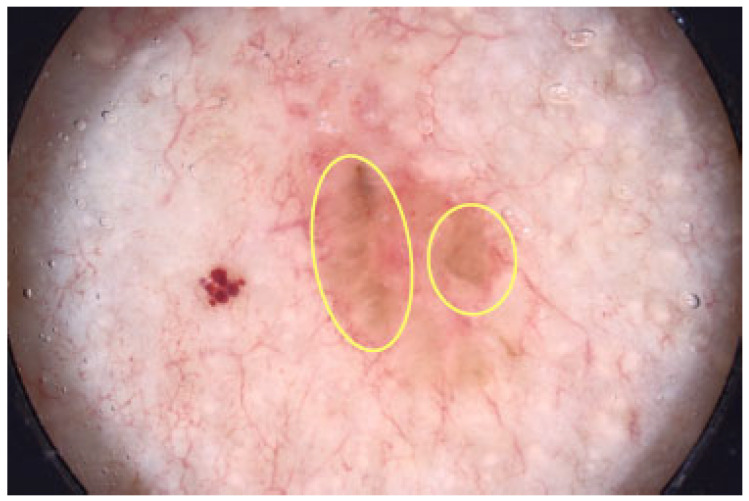
Dermoscopy image of BCC with brown homogeneous blotches (BHB) (yellow circles).

In 2023 Manca et al. reported presence of patches of uniform brown pigmentation without any other dermoscopic structures, except for occasionally present arborizing vessels or globules/dots, in 61 of 90 cases of pigmented BCC. Based on this, the authors introduced “brown homogeneous blotches” as a new dermoscopic finding in pigmented BCC. They demonstrated a sensitivity of 67.8% and a specificity of 93.3%. These values were comparable with those of well-known dermoscopic criteria of pigmented BCC. BHB showed an even higher sensitivity than some already established features, such as concentric structures (21.1%), spoke-wheel areas (6.7%) and MLLAs (32.2%) [3]. Figure 18 presents BCC with BHB.

##### Large Blue-Gray Structureless Areas

**Figure 19:**
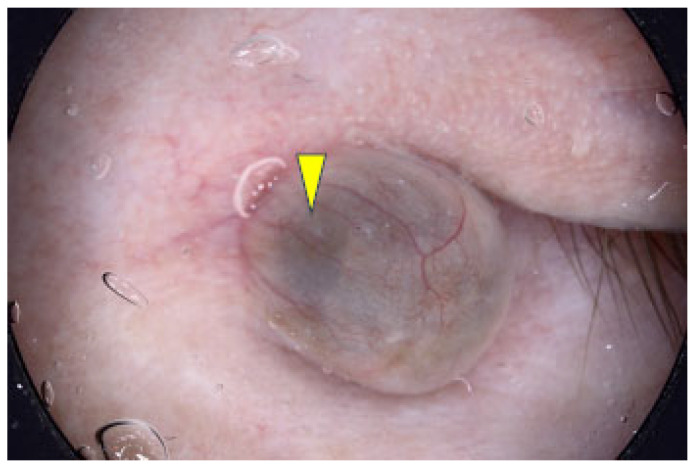
Dermoscopy image of BCC with large blue-gray structureless areas (yellow arrowhead).

In a study from 2021 evaluating the relationship between BCC size and dermoscopic features, a high incidence (56%) of diffuse blue-gray patches was noted in large BCCs. The authors suggested that this finding results from the integration of large blue-gray ovoid nests and may serve as an important clue for identification of large BCCs [55]. An example of BCC with large blue-gray structureless areas is presented in Figure 19.

##### Rainbow Pattern

In polarized dermoscopy, as mentioned by Garcia-Garcia and Perez-Oliva in 2010, BCC may present with various colors of the rainbow spectrum (ranging from red to violet). However, this multicolored pattern has been predominantly observed in Kaposi sarcoma, melanoma, stasis dermatitis and lichen planus [56].

##### Semitranslucent Areas

**Figure 20:**
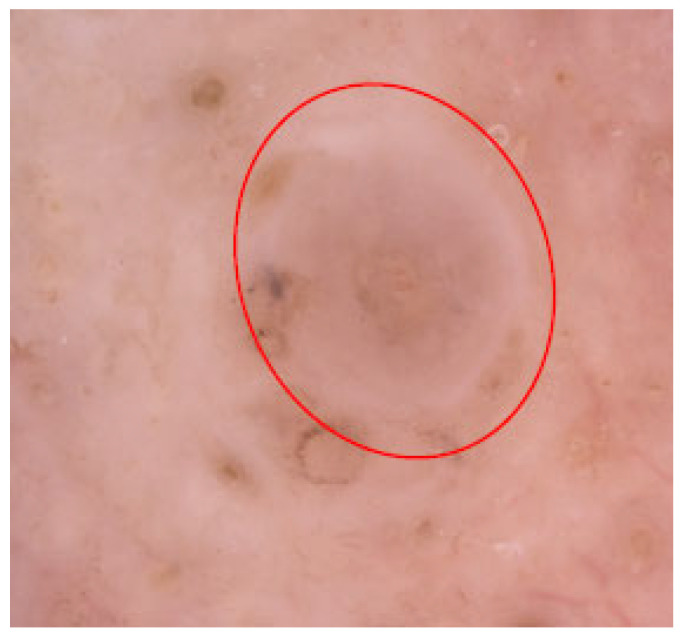
Dermoscopy image of BCC with semitranslucent areas (red circle).

In 2009 Stoecker et al. observed multiple colors in BCC, ranging from reddish pink in thicker regions, dull orange at the periphery, to occasionally gray areas, which the authors termed semitranslucent areas. This phenomenon is best seen under non-contact polarized dermoscopy. While contact polarized imaging may reduce the typical color, it preserves the contrast in smoothness with the surrounding areas, which is key to this “jelly-like” phenomenon. This feature was found to correlate in histopathology with basaloid tumor nodules located near the surface, as well as with reduced epidermal thickness and a thinner collagen layer [57]. The BCC presenting this feature is shown in Figure 20.

##### Interrupted Radial Streaking

In a single case reported by Bakos et al. in 2007, a patient with phototype IV presented with superficial pigmented BCC that exhibited numerous long and short pigmented streaks, ranging from brown to black. These streaks were arranged in an interrupted radial and centrifugal pattern, occasionally fusing together, creating an interrupted pigment network, which could easily be misdiagnosed as melanoma [47].

The summary of dermoscopic findings seen in BCC is presented in Table 1.

## 4. Discussion

The current review highlights the high diagnostic accuracy of dermoscopy in identifying BCC when compared with clinical examination alone. Dermoscopy has proven especially valuable in distinguishing pigmented and non-pigmented variants of BCC. The studies show high sensitivity (ranging from 67.6% to 98.6%) and PPV (ranging from 85.9% to 97%) in the detection of BCC. The high specificity (up to 100% for malignant skin lesions in general) underscores dermoscopy’s reliability as a diagnostic tool for skin malignancies.

However, some challenges remain. Diagnostic sensitivity appears to be lower for lesions located on the trunk and extremities, particularly those of a superficial subtype, which are often hypopigmented or amelanotic. This highlights the importance of considering lesion-specific characteristics in dermoscopic analysis. Studies also show that the experience and training of the physician significantly improve diagnostic accuracy.

In terms of dermoscopic features, BCC presents a wide range of patterns. Pigmented BCCs often show features like large gray-blue ovoid nests, multiple gray-blue globules and arborizing vessels, while non-pigmented BCCs typically display vascular patterns like arborizing vessels and fine telangiectasias. The presence of shiny white lines, seen with polarized light, is an important sign of malignant lesions, including BCC and melanoma. Additionally, structures like MAY globules have been linked to more aggressive BCC subtypes, helping with risk assessment.

The limitation of the current review is that the search was restricted to the PubMed database. Additionally, a limitation in the literature is the need for histopathological confirmation to accurately predict aggressive BCC subtypes. While some studies suggest dermoscopy can predict these subtypes with over 93% accuracy, particularly for superficial and nodular variants, aggressive BCC subtypes are harder to diagnose dermoscopically, perhaps because they invade deeper. Further research is needed to identify specific dermoscopic features that can reliably indicate these more challenging cases.

Some recently identified dermoscopic features, like NMLLAs, brown homogeneous blotches and large blue-gray structureless areas, show promise but need more validation. Although these features may improve diagnostic accuracy, their role in routine practice has yet to be clearly defined.

## 5. Conclusions

In summary, dermoscopy is a noninvasive, highly accurate method for diagnosing BCC, with distinct dermoscopic features that are associated with both pigmented and non-pigmented subtypes. While the accuracy of dermoscopy is particularly high for pigmented BCC, there are ongoing challenges with non-pigmented lesions. Future research should focus on standardizing dermoscopic criteria for aggressive BCC variants and further exploring newer dermoscopic features. These efforts will enhance the effectiveness of dermoscopy in diagnosing BCC.

## Figures and Tables

**Table 1:** A summary of dermoscopic findings in basal cell carcinoma (BCC).

Dermoscopy Finding	Definition	Mechanism of Formation and Corresponding Pathological Findings	Remarks
Shiny white lines (‘crystalline structures’ or ‘crystalline lines’)	Bright whitish lines visible only under polarized light; include short lines and longer strands; longer strands are typically arranged in parallel or disorganized; more frequently present in BCCs with ulceration	Interaction of polarized light with collagen orientation in the stromal tissue of tumor characterized by elevated amount of dermal collagen [25]	Higher risk of malignancy; reliable criterion for detecting BCC; other lesions include melanoma, SCC, dermatofibromas, scars, sun-damaged skin
Shiny white areas (blotches)	Large structureless white areas seen only under polarized light;	Interaction of polarized light with collagen orientation in the stromal tissue of tumor characterized by elevated amount of dermal collagen [25]	Reliable criterion for detecting BCC
Rosettes	Four bright white points seen only under polarized light	Optical phenomenon caused by the interaction of polarized light with keratin-filled adnexal openings [26]	Not typically associated with BCC, more common in AK and SCC
Milia-like cysts (MLCs)	Starry (bright center, variably sharp borders) or cloudy (larger, fluffy borders, oval in shape) yellow or white formations; more clearly visible under non-polarized dermoscopy	No correlation with pathology was found in the literature analyzed in the study	Not specific to BCC (typically in seborrheic keratosis and congenital melanocytic nevi); their presence should not rule out a BCC diagnosis when other specific dermoscopic criteria are present;
Yellow lobular-like structures	Round or oval yellow structures	No correlation with pathology was found in the literature analyzed in the study	More common in BCCs on the face and nodular BCC; characteristic of sebaceous hyperplasia, sebaceous adenoma, nevus sebaceous of Jadassohn
MAY globules	Clustered white-yellow structures; visible under polarized and non-polarized light	Localized, circular regions of abnormal calcification within or surrounding tumor masses, accompanied by calcified keratocysts [32]	High-risk histologic subtypes of BCC (infiltrative, morpheaform, micronodular); helpful in distinguishing BCC from intradermal nevi, excluding the latter; present also in SCC or desmoplastic trichoepithelioma
Arborizing vessels	Large vessels branching in a tree-like pattern; more clearly visible in non-contact dermoscopy	Main vessels measuring ≥0.2 mm in diameter with irregular, tree-like branching [30]; Arborizing microvessels indicate telangiectasia smaller than 0.2 mm in diameter [42]	The most common BCC feature; associated with nodular, pigmented and non-ulcerated BCC; In non-BCC, the number of ramifications was lower than in BCC, and the diameter of vessels decreased more acutely from the stem vessel to the first branch
Short fine telangiectasias	Small kinked vessels of small caliber; an early form of arborizing vessels; more clearly visible in non-contact dermoscopy	Thin, twisted vessels of small diameter and short length, with minimal branching [36]	Associated with superficial and non-pigmented BCC
Multiple small erosions/ulcerations	Erosion—superficial tissue loss; ulceration—loss of the entire epidermis and superficial dermis	Erosion—superficial tissue loss; ulceration—loss of the entire epidermis and superficial dermis [41]	Multiple small erosions are characteristic for superficial BCC
Regression features (“blue areas”, “blue hue”, “pepper-like structures, “white scar-like areas”, “white areas”, “milky way areas”)	Bluish or white/reddish areas indicating melanin accumulation (bluish) or fibroplasia with the formation of blood vessels (white/reddish)	“Blue areas”, “blue hue” and “pepper-like structures” correspond to increased melanin deposition in the dermis. “White areas”, “white scar-like areas” and the “milky way areas” indicate fibroplasia associated with blood vessel formation, leading to a whitish appearance with varying reddish shades [42]	Regression features disrupt the overall dermoscopic presentation of the lesion, making the diagnosis more challenging; should not be considered an independent indicator of melanoma
Pigment network	Intersecting brown lines creating a reticular pattern with hypopigmented holes	Melanin present within keratinocytes and/or melanocytes along the junction of the epidermis and dermis [42]	Proves against the diagnosis of BCC; mostly associated with the collision of BCC with another skin neoplasm (e.g., solar lentigo, nevus or actinic keratosis) or lesion’s location on photodamaged skin
Spoke-wheel areas	Radial brown, blue or gray projections converging at a central axis darker in color	Clusters of pigmented basaloid cells extending from the follicular epithelium [42]	The most significant factor in predicting pigmented BCC; most common in thinner tumors
Maple leaf-like areas (MLLAs)	Brown to gray-blue bulbous extensions; do not arise from a pigment network and an adjacent confluent pigmented area	Extensive, complex masses of pigmented basaloid cells located in the upper dermis [42]	May indicate early stage pigmented BCC
Large gray-blue ovoid nests	Large ovoid pigmented areas not directly connected to the main tumor body	Prominent clusters of pigmented basaloid cells within the dermis [42]	Associated with increased thickness of pigmented BCC; higher risk of malignancy; rarely observed also in nevi, seborrheic keratoses or angiomas
Multiple blue/gray globules	Multiple globules blue or gray in color	Compact clusters of pigmented basaloid cells found in the dermis [42]	Classic dermoscopic features of pigmented BCC
Multiple in-focus blue/gray dots	Foci of multiple blue/gray dots that appear “in focus” at dermoscopic examination	Smaller than in globules clusters of melanocytes or melanin granules in the papillary dermis [42]	Indicate the early phase of multiple blue/gray globules in pigmented BCC
Blue-whitish veil	Blue structureless zone; better seen in non-polarized dermoscopy	Brown pigment deposition in the dermis consisting of melanin-laden melanocytes and/or melanophages, with orthokeratosis, hypergranulosis and occasional parakeratosis above the pigment [51]	Traditionally associated with melanoma, however also frequently seen in pigmented BCC
Brown dots/globules	Dots or globules brown in color	Smaller clusters (dots) or larger clusters (globules) of melanocytes or melanin granules at the dermoepidermal junction [42]	Proves against the diagnosis of BCC (typical for melanocytic lesions)
Concentric structures	Irregularly shaped globular-like structures with different colors (blue, gray, brown, black) and darker central area	No correlation with pathology was found in the literature analyzed in the study	Represent the early stage of a spoke-wheel area in pigmented BCC
Negative maple leaf-like areas (NMLLAs)	Non-pigmented version of the MLLAs	Non-pigmented tumor clusters at the dermal-epidermal junction [2]	Superficial BCC
Brown homogeneous blotches	Patches of uniform brown pigmentation	The study reporting on BHB did not provide any correlation with pathology [3]	Pigmented BCC
Large blue-gray structureless areas	Diffuse blue-gray patches	The authors who first reported on this feature suggested that it results from the integration of large blue-gray ovoid nests; however, no correlation with pathology was assessed [55]	Important clue for identification of large BCC
Interrupted radial streaking	Brown or black streaks arranged in an interrupted radial and centrifugal pattern	Pigmented multicentric superficial BCC with melanophages present in the fibrotic upper dermis [47]	Can be confused with melanoma
Rainbow pattern	Multicolored pattern seen under polarized dermoscopy	The study reporting on rainbow pattern did not provide any correlation with pathology [56]	Rare in BCC, more common in other entitites (e.g., Kaposi’s sarcoma, melanoma, stasis dermatitis, lichen planus)
Semitranslucent areas	Jelly-like phenomenon with reddish pink or gray color, seen under non-contact polarized dermoscopy	Basaloid tumor nodules located near the surface, reduced epidermal thickness and a thinner collagen layer [57]	In BCC correlates with basaloid tumor nodules located near the surface, reduced epidermal thickness and a thinner collagen layer

## Data Availability

No original datasets were generated for this article.
